# Gems of Pediatric Dermatology: Response to Burshtein et al, “Efficacy, perception and utilization of pediatric teledermatology: A systematic review”

**DOI:** 10.1016/j.jdin.2023.11.003

**Published:** 2023-11-30

**Authors:** Mia A. Mologousis, Diana Olvera, Elena B. Hawryluk

**Affiliations:** a Tufts University School of Medicine, Boston, Massachusetts; b Dermatology Program, Boston Children’s Hospital, Boston, Massachusetts; c Department of Dermatology, Massachusetts General Hospital, Boston, Massachusetts; d New York Institute of Technology College of Osteopathic Medicine, Jonesboro, Arkansas; e Harvard Medical School, Boston, Massachusetts

**Keywords:** infantile transient smooth muscle contraction of the skin, pediatric dermatology, sock-line hyperpigmentation, telemedicine, transient abdominal telangiectasia of the newborn

*To the Editor:*

The published manuscript from Burshtein et al^1^ regarding pediatric teledermatology provided great insight into how virtual care has expanded access within a field facing a chronic clinician shortage. One additional benefit of telemedicine not captured by statistics is its exposure of clinicians to rare and transient conditions. We share 3 cases in which telemedicine enabled prompt diagnosis in patients who otherwise may not have had subspecialist access, or who may have undergone unnecessary workup.

A healthy 5-month-old boy experienced episodic, asymptomatic rippling of the left thigh skin. Flares were not reproducible in his pediatrician’s office. However, history and photos presented during a teledermatology encounter facilitated his diagnosis with infantile transient smooth muscle contraction of the skin (ITSMC). ITSMC, described in 2013, is a rare poorly-understood phenomenon, likely resulting from transient contraction of the skin’s arrector pili smooth muscle fibers secondary to autonomic immaturity, primitive reflexes, or smooth muscle hypersensitivity.^2^ ITSMC resolves completely between episodes and is not associated with hyperpigmentation or hypertrichosis, making the diagnosis challenging.^3^

A full-term infant with self-resolved neonatal jaundice presented with telemedicine photos at age 16 days demonstrating bright red telangiectatic vessels on the abdomen, clustered on each side. Photos and later live clinic evaluation confirmed the diagnosis of transient abdominal telangiectasia of the newborn (TATN). TATN, coined in 2021, describes rare, self-limited, and symmetric “butterfly wing” telangiectasias on the neonate’s abdomen within 1 month of life.^3^ It is hypothesized that intraabdominal pressure disrupts the skin’s venous return, causing telangiectasias that can be confirmed via dermatoscopy.^3^ TATN typically resolves before age 3 months, providing a limited window for in-person evaluation. Prompt diagnosis enabled avoidance of laboratory and ultrasonographic workup and provided an expedited live visit to confirm diagnosis and educate trainees.

A healthy full-term infant presented via telemedicine at age 3 months. Photographs demonstrated a linear red patch on the foot noted at age 1 month. From appearance and literature review, we suspected a diagnosis of “sock line bands,” confirmed at live appointment at age 5 months. At that live visit, the patch had faded significantly. Described in 2021, infantile garment bands have been described in a handful of reports following sock, mitten, pant, and diaper distributions.^4^^,^^5^ Garment constriction may cause dermal inflammation and postinflammatory changes that improve over months to years. Differential diagnosis includes amniotic bands, acquired raised bands of infancy, and child abuse; recognition can therefore prevent unnecessary diagnostic workup and provide reassurance.

In the same way that telemedicine improves patient access to subspecialist pediatric dermatologists, it also allows the pediatric dermatologists access to rare conditions that may never otherwise be seen in clinic. Telemedicine permits triaging of interesting and rare diagnoses to enhance teaching. Historically, children presented without findings, and parents struggled to describe what they had seen; opportunities to diagnose and even examine rare and transient diseases were slim. In addition to Burshstein et al’s comprehensive discussion, teledermatology is just as valuable a resource for us clinicians as it is for patients and parents seeking our care.

## Conflicts of interest

Dr Hawryluk is an author and reviewer for UpToDate, serves as a consultant for Skin Analytics, and has received research materials from DermTech. Authors Mologousis and Olvera have no conflicts of interest to declare.
